# Associations between Depression and Diabetes in the Community: Do Symptom Dimensions Matter? Results from the Gutenberg Health Study

**DOI:** 10.1371/journal.pone.0105499

**Published:** 2014-08-15

**Authors:** Jörg Wiltink, Matthias Michal, Philipp S. Wild, Astrid Schneider, Jochem König, Maria Blettner, Thomas Münzel, Andreas Schulz, Matthias Weber, Christian Fottner, Norbert Pfeiffer, Karl Lackner, Manfred E. Beutel

**Affiliations:** 1 Department of Psychosomatic Medicine and Psychotherapy, University Medical Center of the Johannes Gutenberg-University Mainz, Mainz, Germany; 2 Center for Thrombosis and Hemostasis, University Medical Center of the Johannes Gutenberg-University Mainz, Mainz, Germany; 3 Department of Medicine 2, University Medical Center of the Johannes Gutenberg-University Mainz, Mainz, Germany; 4 German Center for Cardiovascular Research (DZHK), partner site RhineMain, Mainz, Mainz, Germany; 5 Institute of Medical Biostatistics, Epidemiology and Informatics, University Medical Center of the Johannes Gutenberg-University Mainz, Mainz, Germany; 6 Department of Medicine 1, University Medical Center of the Johannes Gutenberg-University Mainz, Mainz, Germany; 7 Department of Ophthalmology, University Medical Center of the Johannes Gutenberg-University Mainz, Mainz, Germany; 8 Institute of Clinical Chemistry and Laboratory Medicine, University Medical Center of the Johannes Gutenberg-University Mainz, Mainz, Germany,; Institute of Psychiatry, United Kingdom

## Abstract

**Objectives:**

While a bidirectional relationship between diabetes and depression has been established, there is little knowledge if the associations are due to somatic-affective or cognitive-affective dimensions of depression.

**Research Design and Methods:**

In a population-based, representative survey of 15.010 participants we therefore studied the associations of the two dimensions of depression with diabetes and health care utilization among depressed and diabetic participants. Depression was assessed by the Patient Health Questionnaire PHQ-9.

**Results:**

We found a linear and consistent association between the intensity of depression and the presence of diabetes increasing from 6.9% in no or minimal depression to 7.6% in mild, 9% in moderate and 10.5% in severe depression. There was a strong positive association between somatic-affective symptoms but not with cognitive-affective symptoms and diabetes. Depression and diabetes were both independently related to somatic health care utilisation.

**Conclusions:**

Diabetes and depression are associated, and the association is primarily driven by the somatic-affective component of depression. The main limitation of our study pertains to the cross-sectional data acquisition. Further longitudinal work on the relationship of obesity and diabetes should differentiate the somatic and the cognitive symptoms of depression.

## Introduction

With an increasing prevalence diabetes has become a significant public health burden (e.g. [1]). About 10 percent of diabetes patients also suffer from clinically significant depressive symptoms, and between 25 and 30 percent reported subclinical depressive symptoms (e.g. [2]–[4]).

There is evidence from longitudinal studies that depressive symptoms contribute to the incidence of diabetes in addition to obesity or antidepressant drug use (e.g. [5]). A recent meta-analysis based on 23 studies with more than 400.000 participants and a mean follow-up of 8.3 years by Rotella & Mannucci [6] found that the yearly incidence of diabetes was higher in the initially depressed compared to the nondepressed participants (0.72 vs. 0.47%).

Several meta-analyses on longitudinal data have indicated a bidirectional relationship between diabetes and depression, however, evidence for the direction that diabetes causes depression is somewhat weaker (e.g. [7], [8]; for overview: [9], [10]). Comorbid depression is consistently related to diabetes complications (diabetic retinopathy, nephropathy, neuropathy, macrovascular complications, and sexual dysfunction) [11] and also to treatment nonadherence [12]. Insulin-dependent diabetes patients have to cope with specific problems as difficulties with the integration of treatment into daily live, self-responsibility, dietary restrictions, or fear of complications, all contributing to depression and reducing medication adherence and therefore impair regulation of blood glucose leading to diabetes complications in later life. In addition, adverse health behavior (e.g. smoking, physical inactivity) associated with depression may compound diabetic complications [13].

Most studies focused on depressive symptoms in general as indicated e.g. by the PHQ sum score [14] or diagnostic interviews. For different somatic conditions (e.g. cardiovascular disease, obesity) only the somatic-affective symptoms were associated with poor medical outcome [15]–[18]. Studies differentiating cognitive-affective and somatic-affective depressive symptoms in diabetes are rare (e.g. [19], [20]). To our knowledge, no study to date has investigated the associations of somatic-affective and cognitive-affective symptoms to diabetes in a large population based study.

In our cross-sectional population based sample we sought to answer the following issues:

1. Are intensity and symptomatology of depression associated to diabetes?
2. How are depression and diabetes associated to the utilization of mental and somatic health care?

We expected an increased prevalence of depressive symptoms among diabetic participants. We also expected a closer relationship between diabetes and somatic-affective depressive symptoms rather than cognitive-affective depressive symptoms. We further expected more frequent health care utilization and psychopharmacological treatments in the participants suffering from both diabetes and depressive symptoms.

## Materials and Methods

### Procedure and study sample

We investigated cross-sectional data of N = 15.010 participants enrolled in the Gutenberg Health Study (GHS) from 2007 to 2012. The GHS is a population-based, prospective, observational single-center cohort study in the Rhine-Main-Region in western Mid-Germany. The GHS has been approved by the local ethics committee and by the local and federal data safety commissioners. The primary aim of the study is to evaluate and improve cardiovascular risk stratification. The sample was drawn randomly from the local registry in the city of Mainz and the district of Mainz-Bingen. The sample was stratified 1∶1 for gender and residence and in equal strata for decades of age. Inclusion criteria were age 35 to 74 years and written informed consent. Persons with insufficient knowledge of German language, or those who reported that they were not able to visit the study center on their own (due to their physical and/or mental condition) were excluded. The response rate (defined as the recruitment efficacy proportion, i.e. the number of persons with participation in or appointment for the baseline examination divided by the sum of number of persons with participation in or appointment for the baseline examination plus those with refusal and those who were not contactable) was 60.3% for the first 5.000 participants. Due to the ongoing recruitment of the GHS, which is conducted in waves, a final statement concerning the response rate cannot be made at this time. The design and the rationale of the Gutenberg Health Study (GHS) have been described in detail elsewhere [21].

### Materials and Assessment

The 5-hour baseline-examination in the study center comprised evaluation of prevalent classical cardiovascular risk factors and clinical variables, a computer-assisted personal interview, laboratory examinations from a venous blood sample, blood pressure and anthropometric measurements. In general, all examinations were performed according to standard operating procedures (SOPs) by certified medical technical assistants.

### Primary outcome measures

#### Depression

Depression was measured by the Patient Health Questionnaire (PHQ-9); caseness was defined by a score ≥10 with a sensitivity of 81% and a specificity of 82% for depressive disorder (14). Depressive symptoms were classified as “minimal” (score 5 to 9), “mild” (score 10 to 14), “moderately severe” (score 15 to 19) and “severe” (score >20) [22]. The somatic-affective and cognitive-affective dimensions of depression were defined according to prior studies [23]–[25]. Four PHQ-9 items related to problems with sleep, fatigability, appetite, and psychomotor agitation/retardation were classified as somatic-affective symptoms, whereas 5 items, related to lack of interest, depressed mood, negative feelings about self, concentration problems and suicidal ideation, were classified as cognitive-affective symptoms of depression [16], [18]. While we were aware, that dimensions of depression (cognitive-affective and somatic-affective) in the community might differ from those in cardiovascular settings, we used the same dimensions for comparison purposes and due to their high face validity and comparability.

#### Diabetes

*Diabetes* was defined in individuals with a definite diagnosis of diabetes by a physician or a blood glucose level of ≥126 mg/dl in the baseline examination after an overnight fast of at least 8 hours or a blood glucose level of ≥200 mg/dl in the baseline examination after a fasting period <8 hours.

#### Health care utilization

We determined two variables indicating health care utilization regarding mainly somatic or mainly mental problems. We assessed whether subjects had *consulted somatic physicians* (general practitioner or medical specialists) and whether they had *consulted psychotherapists/psychiatrists* during the last 4 weeks.

#### Potential confounders

In addition to age and sex we predefined a comprehensive set of confounders with a potential relation to diabetes and/or depression.

#### Life style factors

*The socioeconomic status* (SES) was defined according to Lampert's and Kroll's Scores of SES range from 3 to 27 while 3 indicates the lowest SES and 27 the highest SES [26].

#### Anxiety

*Generalized anxiety* was assessed with the two screening items of the short form of the GAD-7 (Generalized Anxiety Disorder [GAD] – 7 Scale) [27], [28]. A sum score of 3 and more (range 0–6) out of these two items indicates generalized anxiety with good sensitivity (86%) and specificity (83%) [28]. *Panic disorder* was screened with the brief PHQ panic module. Caseness was defined if at least two of the first four PHQ panic questions are answered with “yes” [29].The German version of the Mini-Social Phobia Inventory (Mini-Spin) [30] was used to detect *social anxiety*. Utilizing a cut-off score of 6 (range 0–12), the Mini-Spin is supposed to separate between individuals with generalized social anxiety disorder and controls with good sensitivity (89%) and specificity (90%) [30], [31]. Suffering from *any anxiety* was defined by reaching the cut-off in at least one of the three above mentioned scales [31].

#### Somatic conditions

*Hypertension* was diagnosed, if antihypertensive drugs were taken, or a mean systolic blood pressure of ≥140 mmHg in the 2^nd^ and 3^rd^ standardized measurement after 8 and 11 minutes of rest or a mean diastolic blood pressure of ≥90 mmHg in the 2^nd^ and 3^rd^ standardized measurement after 8 and 11 minutes of rest. *Obesity* was defined as a Body-Mass-Index (BMI) ≥30 kg/m^2^. *Dyslipidemia* was defined as a definite diagnosis of dyslipidemia by a physician or an LDL/HDL-ratio of >3.5. The presence of further somatic conditions was assessed within a structured interview: “Has a physician ever diagnosed: constriction of your coronary heart vessels (*coronary heart disease,* CHD), *atrial fibrillation*, *cancer*, *myocardial infarction*, or *stroke*?”

#### Psychotropic medication

The following *psychotropic medications* potentially affecting mood and/or metabolism were chosen as confounders: non-selective monoamine reuptake inhibitors, selective serotonin reuptake inhibitor, other antidepressants, antipsychotics, anxiolytics, hypnotics/sedatives, antiepileptics, opioids.

### Statistical analysis

Statistical analysis was done by IBM SPSS Statistics 20 (IBM, Chicago, IL).

Data are presented as numbers/percentage, mean (and 1.96-fold standard deviation) or median (and 1^st^, 3^rd^ quartile) as appropriate.

To determine prevalence rates of diabetes stratified by severity of depressive symptoms we weighted our data by age and gender based on the population in the region of Mainz/Mainz-Bingen.

Odds ratios of single items differentiating the diabetic and non-diabetic populations were computed by ordinal logistic regression analyses (cumulative logit) of the PHQ-9 items on diabetes status. The models were adjusted by age, sex and SES.

To analyse the relationship between depression and diabetes, we computed separate linear regression models with depression (PHQ-9 sum score) as the dependent variable. Model 1 was without adjustment; in model 2 we adjusted for age, gender and socioeconomic status (SES) as potential confounders of depression and diabetes. Depressive symptoms were additionally evaluated with two separate analyses: a) using somatic-affective symptoms of depression (PHQ somatic-affective sum score) and b) using cognitive-affective symptoms (PHQ cognitive-affective sum score) as dependent variables. For these analyses we additionally adjusted (model 3) for cognitive-affective symptoms (dependent variable: somatic affective symptoms) and for somatic-affective symptoms (dependent variable: cognitive affective symptoms). Dependent variables were transformed in order to optimize the regression model: ln(PHQ-9 sumscore +5), ln(somatic-affective sum score +5), ln(cognitive-affective sum score +2), where ln denotes the natural logarithm.

To determine relations between depression (caseness: PHQ sum score <10 vs. PHQ sum score > = 10), diabetes and health care utilization we used logistic regression models with the dichotomous variables a) *consultation of somatic physicians* and b) *consultation of psychotherapists/psychiatrists* as the dependent variables and diabetes, depression and their interaction term (diabetes × depression) as independent variables. Models were adjusted for age, gender, socioeconomic status (SES), any anxiety and somatic conditions. In order to reduce total number of predictors we performed a variable selection for somatic conditions (obesity, hypertension, dyslipidemia, atrial fibrillation, CHD, myocardial infarction, stroke, cancer). Only those somatic conditions significantly related to health care utilization (logistic regression model with backward enter procedure) were entered into our regression. For the visualization of the results of these logistic regression models we computed model based estimates of marginal population means for each of the four groups defined by presence or absence of diabetes and depression.

All p-values correspond to 2-tailed tests.

## Results

### Sample characteristics

Table 1 shows the sociodemographic characteristics (age, sex, SES), depressive symptoms, anxiety, psychotropic medication, somatic conditions and health care utilization stratified for severity of depressive symptoms (no/minimal to moderately severe/severe).

**Table 1:** Sample characteristics stratified for severity of depressive symptoms (N = 14.731).

	PHQ score			
	0-4-5	5–9	10–14	15–27
Number	9566	4032	856	277
Sex, female	45.3%	56.6%	59.9%	61.4%
Age, in years; mean (SD)	55.3 (11.2)	54.5 (11.0)	53.2 (10.3)	51.6 (9.8)
SES, (3-21), median (1^st^, 3^rd^ quartile)	13 (10/17)	12 (9/16)	11 (9/15)	11 (8/14)
Depressive symptoms				
Somatic-affective, median (1^st^/3^rd^ quartile)	1 (1/2)	4 (3/4)	6 (5/7)	9 (7/10)
Cognitive-affective, median (1^st^,/3^rd^ quartile)	0 (0/1)	3 (2/4)	5 (4/6)	9 (8/10)
Any Anxiety	3.4%	18.5%	55.2%	86.1%
Psychopharmacological treatment	4.8%	12.3%	24.0%	41.5%
Somatic conditions				
Diabetes	6.9%	7.6%	9.0%	10.5%
Diabetes untreated/unaware	0.7%	0.4%	0.5%	0.4%
Obesity (BMI> = 30)	23.2%	27.2%	30.5%	38.8%
Hypertension	50.1%	48.7%	47.3%	49.6%
Dyslipidemia	28.6%	29.9%	34.2%	35.4%
Atrial fibrillation	2.6%	2.9%	2.4%	3.3%
CHD	4%	4.7%	5.5%	5.8%
Myocardial infarction	2.6%	3.5%	3.0%	3.3%
Stroke	1.6%	2.0%	3.1%	1.8%
Cancer	8.5%	10.2%	10.2%	7.2%
> three somatic conditions	3.0%	4.0%	5.1%	5.7%
Health care utilization (last 4 weeks)				
Consulted somatic physicians	39.4%	47.1%	57.9%	64.6%
Consulted psychotherapists/psychiatrists	0.2%	0.4%	2.1%	9%

The mean age of the participants was 55.0 years (range 35–74 years). 7428 were male (50.4%), and 7303 participants were female (49.6%). The majority of the participants reported a low level of education (less than 10^th^ grade). About 1/3 had completed high school.

Prevalence of diabetes (weighted by age and gender) in the group of participants with no or only minimal depressive symptoms was 5.8%, mild depression 6.4%, moderate 7.5% and moderately severe/severe depression 9.1%. There was a considerable proportion of participants with an untreated or undetected (unaware) diabetes (no or only minimal depressive symptoms 0.6%, mild depression 0.4%, moderate depression 0.4% and moderately severe/severe 0.3%). Unawareness among diabetics decreased with increasing depression: no or only minimal depressive symptoms 10.2%, mild depression 6.9%, moderate depression 5.1% and moderately severe/severe 3.4%.

### Associations between depression and diabetes

In our linear regression model, diabetes was significantly positively related to depressive symptoms after controlling for age, gender, socioeconomic status (SES). Using only the somatic-affective symptoms of depression as dependent variable and additionally controlling for the cognitive-affective symptoms, the same picture emerged. Cognitive-affective symptoms of depression were unrelated to diabetes. See table 2.

**Table 2:** Linear regression analyses of diabetes and depression (N = 14631–14639).

	Depressive symptoms (PHQ)	Somatic-affective symptoms	Cognitive-affective symptoms
	B (95% CI)	B (95% CI)	B (95% CI)
Model 1	0.037 (−0.025, 0.100)	0.079 (0.016, 0.141)	−0.016 (−0.078, 0.046)
Model 2	0.108 (0.045, 0.170)	0.138 (0.075, 0.201)	0.048 (−0.015, 0.112)
Model 3	..	0.110 (0.059, 0.161)	−0.032 (−0.083, 0.019)

Model 1: without adjustment; Model 2: adjusted for age, gender and socioeconomic status (SES); Model 3: additionally adjusted for cognitive-affective symptoms (dependent variable: somatic affective symptoms) and for somatic-affective symptoms (dependent variable: cognitive affective symptoms).

**Figure 1:**
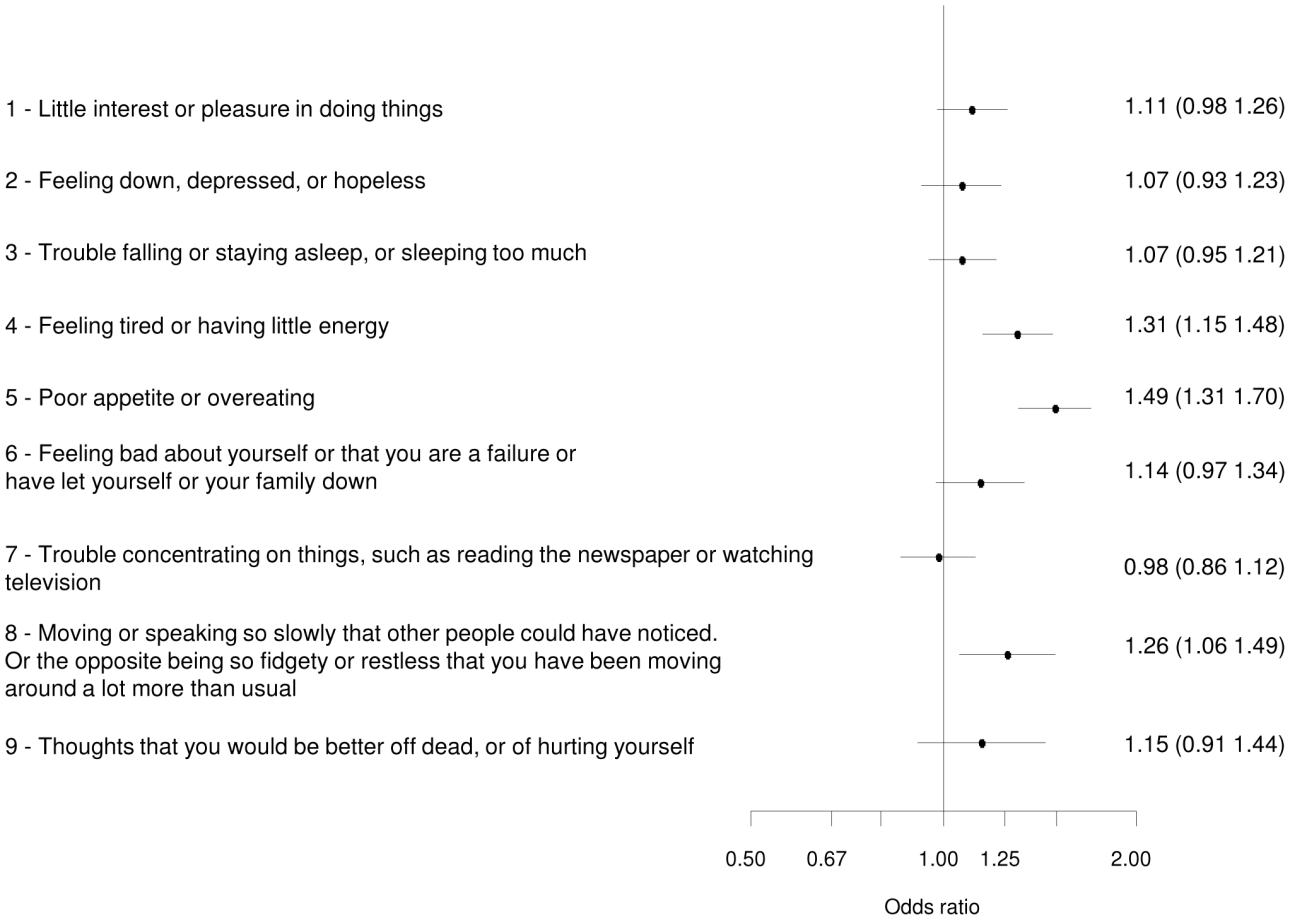
Depressive Symptoms (PHQ): Comparison between the diabetic and non-diabetic population. Ordinal logistic regression analyses (cumulative logit) of PHQ-9 items on diabetes status, models adjusted by age, sex and SES. Odds ratio above 1 indicate a trend towards a higher item response in diabetics. ORs and 95%CI are presented.

Figure 1 shows depressive symptoms for the diabetic and non-diabetic population.

Diabetics scored higher than non-diabetics in the following three items: “Feeling tired or having little energy” (item 4; OR 1.31), “Poor appetite or overeating” (item 5; OR 1.49), and “Moving or speaking so slowly that other people could have noticed. Or the opposite being so fidgety or restless that you have been moving around a lot more than usual” (item 8; OR 1.26). The three items all belong to the somatic-affective symptoms of depression.

### Relation between depression, diabetes and health care utilization

We analysed the relationship between depression (dichotomous; cut-off > = 10), diabetes and health care utilization with logistic regression models.

After controlling for age, gender, SES, substantial symptoms of anxiety, and somatic conditions only depression was significantly related to consultations of psychotherapists/psychiatrists (OR 15.2 [95%CI 9.4, 24.7; p<.001]), diabetes was not (OR 0.70 [95%CI 0.16, 2.98; p = .62]), and neither was the interaction between depression and diabetes (p = .58).

Again, after controlling for the aforementioned variables (age, gender, SES, anxiety, and somatic conditions) depression (OR 1.73 [95%CI 1.48, 2.01; p<.001]) and diabetes (OR 2.00 [95%CI 1.72, 2.33; p<.001]) were significantly related to consultations of somatic physicians, the interaction between depression and diabetes (p = .42) was not.

Results of the logistic regression models (least square means of frequency of health care utilization stratified by depression and diabetes) can be found in Table 3.

**Table 3:** Health care utilization stratified by diabetes and depression (adjusted proportions).

	Consultation of psychotherapists/psychiatrists	Consultation of somatic physicians
	% (95%CI)	% (95%CI)
Depression/Diabetes		
Yes/yes	1.07 (0.25, 4.50)	88.4 (82.1, 92.7)
Yes/no	2.40 (1.41, 4.04)	79.9 (75.9, 83.4)
No/yes	0.14 (0.02, 0.99)	82.1 (78.3, 85.3)
No/no	0.30 (0.17, 0.51)	69.9 (65.4, 74.0)

## Discussion and Conclusions

The principal findings of this paper are a) a strong association between diabetes and depression; b) this was primarily due to somatic-affective rather than cognitive-affective symptoms and c) depression and diabetes were both independently related to somatic health care utilisation.

We found a linear and consistent association of the intensity of depression with the presence of diabetes increasing from 6.9% in no or minimal depression to 7.6% in mild, 9% in moderate and 10.5% in severe depression; i.e. the prevalence of diabetes in severe vs. no depression was elevated substantially (1.5 fold). This finding corresponds to the findings of earlier population based studies (e.g. [2], [4]).

When controlling for comorbid somatic and mental (anxiety) conditions, we found a strong positive association of diabetes with somatic-affective symptoms, but no association with cognitive-affective symptoms. Our findings narrow down the association of depression and diabetes to the somatic-affective dimension. These associations are likely to work both ways: Somatic-affective depressive symptoms are associated with metabolic risk factors for diabetes (obesity, dyslipidemia), which we have demonstrated in a previous paper [16]. Also, metabolic changes in diabetics and the associated inflammation may induce somatic-affective depression (e.g. Sleep disorder, fatigue, increased or decreased appetite, psychomotor changes).

Somatic and mental health care utilization also increased with depression: The majority of severely depressed (64.6% vs. 39.4% no depression) had consulted a physician in the past 4 weeks, and 9% (vs. 0.2%) had consulted a psychiatrist or a psychotherapist. Depressive symptoms were mostly treated pharmacologically, and depression was also associated with psychotherapeutic health care, but to a lesser degree. Taking comorbid conditions into account and controlling for other somatic diseases and anxiety, somatic health care utilization was predicted independently both by depression and by diabetes, i.e. both depressed and diabetic participants had a high somatic health care utilization. However, the lack of an interaction implies that depressive diabetics do not receive more intensive medical treatment than non-depressive diabetics, although it has been demonstrated that depression increased complications and mortality in diabetics [11], [32]. This may indicate an under-supply in those critical patients. As the success of collaborative health care projects suggests [33], [34], depressed patients with comorbid somatic disorders may need more communication between somatic and mental health practitioners.

The main limitation of our study pertains to the cross-sectional data acquisition. Therefore, causal inferences are not possible. Due to the fact that people with less severe complaints are more likely to take part in a community study selection bias might have occurred towards oversampling those with the less severe depressive symptoms. Therefore, our results might not be generalizable to persons with major depressive disorders. Also, we relied on data of validated questionnaires, however, we could not use expert clinical ratings of depression.

The strengths are a) the well characterized, representative sample of participants living in the Rhine-Main region in Germany b) the inclusion of younger participants starting at the age of 35 years and b) the relatively large sample size.

Further, prospective work on the relationship of obesity and diabetes should also a) differentiate the somatic and the cognitive symptoms of depression in elucidating the mechanisms relating depression and diabetes (e.g. genetics, pro-inflammatory cytokines) including moderators (e.g. change in antidepressive or antidiabetic medication, psychotherapy) and b) target effective interventions to prevent and reduce depression in diabetes patients.
